# Survey of Innate Immune Responses to *Burkholderia pseudomallei* in Human Blood Identifies a Central Role for Lipopolysaccharide

**DOI:** 10.1371/journal.pone.0081617

**Published:** 2013-11-26

**Authors:** Narisara Chantratita, Sarunporn Tandhavanant, Nicolle D. Myers, Sudeshna Seal, Arkhom Arayawichanont, Aroonsri Kliangsa-ad, Lauren E. Hittle, Robert K. Ernst, Mary J. Emond, Mark M. Wurfel, Nicholas P. J. Day, Sharon J. Peacock, T. Eoin West

**Affiliations:** 1 Department of Microbiology and Immunology, Faculty of Tropical Medicine, Mahidol University, Bangkok, Thailand; 2 Mahidol-Oxford Tropical Medicine Research Unit, Faculty of Tropical Medicine, Mahidol University, Bangkok, Thailand; 3 Division of Pulmonary and Critical Care Medicine, Department of Medicine, University of Washington, Seattle, Washington, United States of America; 4 Department of Medicine, Sappasithiprasong Hospital, Ubon Ratchathani, Thailand; 5 Department of Clinical Pathology, Sappasithiprasong Hospital, Ubon Ratchathani, Thailand; 6 Department of Microbial Pathogenesis, University of Maryland, Baltimore, Maryland, United States of America; 7 Department of Biostatistics, University of Washington, Seattle, WA, United States of America; 8 Center for Clinical Vaccinology and Tropical Medicine, Nuffield Department of Clinical Medicine, University of Oxford, Churchill Hospital, Oxford, United Kingdom; 9 Department of Medicine, University of Cambridge, Addenbrooke’s Hospital, Cambridge, United Kingdom; 10 International Respiratory and Severe Illness Center, University of Washington, Seattle, Washington, United States of America; Tulane University School of Medicine, United States of America

## Abstract

*B. pseudomallei* is a gram-negative bacterium that causes the tropical infection melioidosis. In northeast Thailand, mortality from melioidosis approaches 40%. As exemplified by the lipopolysaccharide-Toll-like receptor 4 interaction, innate immune responses to invading bacteria are precipitated by activation of host pathogen recognition receptors by pathogen associated molecular patterns. Human melioidosis is characterized by up-regulation of pathogen recognition receptors and pro-inflammatory cytokine release. In contrast to many gram-negative pathogens, however, the lipopolysaccharide of *B. pseudomallei* is considered only weakly inflammatory. We conducted a study in 300 healthy Thai subjects to investigate the *ex vivo* human blood response to various bacterial pathogen associated molecular patterns, including lipopolysaccharide from several bacteria, and to two heat-killed *B. pseudomallei* isolates. We measured cytokine levels after stimulation of fresh whole blood with a panel of stimuli. We found that age, sex, and white blood cell count modulate the innate immune response to *B. pseudomallei*. We further observed that, in comparison to other stimuli, the innate immune response to *B. pseudomallei* is most highly correlated with the response to lipopolysaccharide. The magnitude of cytokine responses induced by *B. pseudomallei* lipopolysaccharide was significantly greater than those induced by lipopolysaccharide from *Escherichia coli* and comparable to many responses induced by lipopolysaccharide from *Salmonella minnesota* despite lower amounts of lipid A in the *B. pseudomallei* lipopolysaccharide preparation. In human monocytes stimulated with *B. pseudomallei*, addition of polymyxin B or a TLR4/MD-2 neutralizing antibody inhibited the majority of TNF-α production. Challenging existing views, our data indicate that the innate immune response to *B. pseudomallei* in human blood is largely driven by lipopolysaccharide, and that the response to *B. pseudomallei* lipopolysaccharide in blood is greater than the response to other lipopolysaccharide expressing isolates. Our findings suggest that *B. pseudomallei* lipopolysaccharide may play a central role in stimulating the host response in melioidosis.

## Introduction

*B. pseudomallei* is a Gram-negative bacterium and the causative agent of melioidosis, a severe disease of human and animals. It is an environmental saprophyte which can infect humans by inhalation, inoculation and ingestion [1]. *B. pseudomallei* is highly virulent, and is classified as a CDC Tier 1 select agent due to concern about its use as a biothreat agent [2]. Melioidosis is endemic in northeast Thailand and in the northern territory of Australia although sporadic and possibly endemic infections are found throughout every continent [1]. Clinical features of melioidosis are diverse, primarily manifesting as sepsis, pneumonia, and abscesses in several organs. Bacteremia occurs in approximately 50% of all cases. Acute melioidosis is often fatal; the overall mortality of patients with melioidosis is as high as 40% and reaches 90% in severe sepsis cases in northeast Thailand [1,3]. Older age is a risk factor for mortality from melioidosis [4,5].

Host innate immune cells such as macrophages, neutrophils, and dendritic cells express pattern-recognition receptors (PRRs) including membrane-bound toll-like receptors (TLRs) and cytoplasmic nucleotide-binding oligomerization domain (NOD)-like receptors (NLRs) that recognize distinct bacterial pathogen-associated molecular patterns (PAMPs) [6]. Well described PAMPs include lipopolysaccharide (LPS) (typically a TLR4 ligand), lipopeptides (TLR2 ligand), flagellin (TLR5 ligand), and peptidoglycan components (NOD1 and NOD2 ligands) [7]. Previous studies suggest the importance of the innate immune response in the control of infection and pathophysiology of sepsis and mortality in melioidosis. In septicemic melioidosis, there is increased expression of TLR receptors and associated molecules including TLR1, TLR2, TLR4, TLR5, TLR10, CD14, and MD-2 mRNA in leukocytes [8]. Melioidosis patients have elevated pro-inflammatory cytokines interleukin IL-12, IL-18, and IL-15, and IFN-γ. Patients who die from melioidosis have higher levels of IL-6 and IL-8 in plasma than those who survive [9]. 

In Gram-negative sepsis, bacterial LPS is considered to play a pivotal role [10]. However, in experimental *B. pseudomallei* infection LPS has not previously been viewed as a key driver of the innate immune response. A number of animal or *in vitro* studies suggest that *B. pseudomallei* LPS is only weakly inflammatory: it is less pyrogenic than *Salmonella abortus equi* LPS, there is a time lag in cytokine production compared to *Escherichia coli* LPS, and there is reduced cytokine and nitric oxide production compared to *E. coli* LPS or *S. typhi* LPS [11,12]. We and others have previously shown that *B. pseudomallei* LPS is a TLR4 agonist [13,14]. However, TLR4 deficiency is not associated with an altered phenotype in murine melioidosis [8]. *B. thailandensis* is a closely related but less virulent organism to *B. pseudomallei* that does not require strict biocontainment conditions for study. In murine airborne *B. thailandensis* infection, TLR4 facilitates early, but not late bacterial containment, and is not required for survival [15]. While these data indicate that *B. pseudomallei* LPS may not be an essential inducer of the immune response in experimental melioidosis, it is not clear what the role of *B. pseudomallei* LPS is in human melioidosis. Our earlier observation that TLR4 region genetic variants are associated with susceptibility to melioidosis in a cohort of Thai subjects raises the possibility that *B. pseudomallei* LPS is important [16].

We hypothesized that the human innate immune response to *B. pseudomallei* is likely to be dependent on various PAMPs acting via multiple PRRs, including *B. pseudomallei* LPS. A challenge in characterizing the inflammatory response in sepsis is the tremendous variation in duration and manifestation of infection. We therefore designed a large study to investigate the innate immune response to *B. pseudomallei* and purified PAMPS *ex vivo* in a human population at risk for melioidosis. Our investigation provides the largest assessment to date of the human innate immune response induced by *B. pseudomallei* and implicates *B. pseudomallei* LPS as a key driver in this host-pathogen interaction.

## Materials and Methods

### LPS isolation, purification, and analysis

A large-scale *B. pseudomallei* K96243 LPS preparation was isolated using a hot phenol/water extraction method after growth in lysogeny broth (LB) supplemented with 1 mM MgCl_2_ at 37°C [17]. Subsequently, LPS was treated with RNase A, DNase I and proteinase K to ensure purity from contaminating nucleic acids and proteins [18]. The LPS sample was additionally extracted to remove contaminating phospholipids [19] and TLR2 contaminating proteins [20] thus generating a preparation suitable for structural analysis and proinflammatory experiments. We have previously demonstrated the lack of TLR2 activation by *B. pseudomallei* LPS prepared in this fashion [13]. LPS fatty acids were derivatized to fatty methyl esters with 2 M methanolic HCl at 90 °C for 18 hours (Alltech) and quantified by gas chromatography using an HP 5890 series II with a 7673 auto injector. Pentadecanoic acid (10 μg; Sigma) was added as an internal standard [21,22]. 

### Immuno-assays

Three hundred Thai subjects donating blood at the blood donation center at Sappasithiprasong Hospital, Ubon Ratchathani, Thailand were recruited for a blood sample. Subjects were included if they indicated they were between the ages of 18 and 60 years and did not report any history of immunodeficiency or inflammatory conditions, chronic diseases, pregnancy in the past six months, anti-inflammatory medication use in the past week, antibiotic use in the past month, vaccination in the past six months, heavy exercise or alcohol consumption in the past 24 hours, or smoking in the past month. Those who met enrolment criteria gave written informed consent to participate and provided a post-donation blood sample in citrate tubes. A batch of ninety-six well immuno-assay plates were generated by adding 20 μl of innate immune ligands and killed bacteria in appropriate concentrations to each well. Plates were frozen at -80°C until the day of use when they were thawed to 37°C. 380 μl of fresh whole blood anticoagulated with citrate from each subject was mixed 1:1 with RPMI media and added to each well [23]. Final concentrations of stimuli were: whole heat-killed *B. pseudomallei* 1026b 2.5 × 10^6^ CFU/ml and K96243 2.5 × 10^6^ CFU/ml, *Salmonella typhimurium* flagellin 500 ng/ml, Pam3CSK4 100 ng/ml (Invivogen), Pam2CSK4 100 ng/ml (Invivogen), MDP 10 μg/ml (Invivogen), TriDAP 10 μg/ml (Invivogen), *B. pseudomallei* K96243 LPS 10 ng/ml (prepared as described above), *E. coli* O111:B4 LPS 10 ng/ml (List Biologicals), and *S. minnesota* Re595 LPS 10 ng/ml (List Biologicals). Plates were placed on a shaker at 37°C under 5% CO_2_ for 6 hours before begun spun down and the plasma supernatant removed and frozen at -80°C. IL-1ra, IL-1β, IL-6, IL-8, IL-10, TNF-α, G-CSF, and MCP-1 concentrations were subsequently assayed using R&D Systems reagents on a Luminex multiplex bead system. Due to out-of-range values in the multiplex assay, IL-1β concentrations for *B. pseudomallei* were determined by ELISA (BD Biosciences).

### Monocyte stimulations

Peripheral blood mononuclear cells (PBMCs) were isolated from blood obtained from healthy subjects at Harborview Medical Center, Seattle who gave written consent to participate. Blood was drawn into two eight ml Vacutainer CPT tubes (Becton Dickinson). Monocytes were obtained from PBMCs using the Monocyte Isolation Kit II (Miltenyi Biotec). Monocytes were plated in a 96 well polypropylene plate at 50,000 cells/well in RPMI media containing 10% FBS and 1%-l glutamine overnight. The following day cells were treated with inhibitors of the LPS-TLR4 axis follows: polymyxin B (Millipore) 100 μg/ml, TLR4/MD2 neutralizing antibody (mab-htlr4md2, Invivogen) 20 μg/ml or control isotype (mabg1-ctrlm, Invivogen) 20 μg/ml prior to addition of stimulus (30 minutes prior for polymyxin B and one hour prior for the antibodies). Cells were stimulated for four hours with heat killed *B. pseudomallei* K96243 at a bacteria:monocyte ratio of 1:1 or *B. pseudomallei* K96243 LPS 1 ng/ml. Supernatants collected were frozen at -30 °C until they were assayed for TNF-α using DuoSet ELISA (R&D Systems).

### Statistical analyses

Immuno-assay cytokine data were log transformed before analysis and correlations were determined by generating a pairwise correlation coefficient. For monocyte studies, given inter-individual variation in responses, all TNF-α values for each individual were normalized to LPS-induced levels from the same subject, and displayed as relative TNF-α units. Analyses of continuous data were made using the *t* test (for two groups), ANOVA with a Bonferroni post test (for three or more groups), or linear regression as appropriate. Statistical analyses were performed with R (version 2.15.2; GNU project [http://www.r-project.org], Stata v11.2, or Graphpad Prism. Differences were considered significant if the p value ≤ 0.05.

### Ethical approval

Approval of consent procedure and protocol for these studies involving human subjects was obtained from the Ethics Committee of the Faculty of Tropical Medicine, Mahidol University, Bangkok, the Ethical Review Committee for Research in Human Subjects, Sappasithiprasong Hospital, Ubon Ratchathani, Thailand, and the University of Washington Human Subjects Division Institutional Review Board. 

## Results

We stimulated whole blood from 300 healthy Thai subjects with a panel of innate immune ligands and quantified the cytokine response in plasma after six hours. We simultaneously measured the white blood cell count (WBC). 127 subjects (42.3%) were female. The median age was 32.8 years, range 17-60 years. The mean (± SD) WBC was 5,341 ± 1,357 cells/μl. We found no association between WBC and age (grouped by decade), or between WBC and sex.

**Figure 1:**
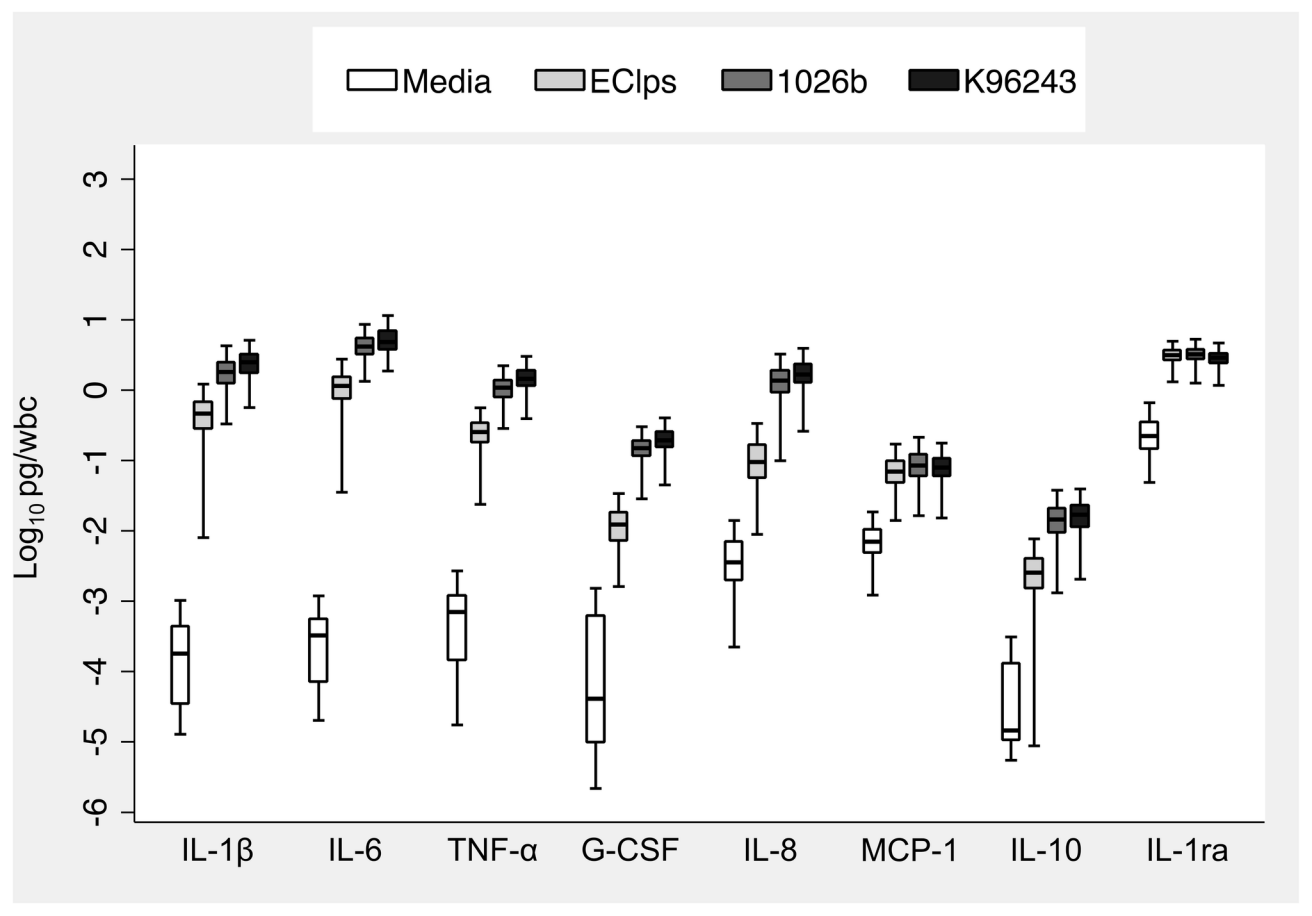
WBC-normalized plasma cytokine concentrations induced by stimulation of whole blood from 300 healthy subjects at 37°C for six hours with medium alone, *E. coli* O111:B4 LPS 10 ng/ml (as a positive control), heat-killed *B. pseudomallei* 1026b 2.5 × 10^6^ CFU/ml, or heat-killed *B. pseudomallei* K96243 2.5 × 10^6^ CFU/ml. Boxes show the median and interquartile range; whiskers show upper and lower adjacent values; outside values are not shown for clarity.

We analysed cytokines induced by stimulation of blood with medium alone or two different heat killed *B. pseudomallei* isolates. Median plasma levels of cytokines induced by medium alone were typically less than 20 pg/ml with the notable exception of IL-1ra (median 1,140 pg/ml). For each stimulant, we observed considerable inter-individual variation in cytokine concentrations of at least one order of magnitude. We investigated the relationship between WBC and cytokine concentration and determined that all eight cytokine responses induced by the two *B. pseudomallei* isolates were highly associated with WBC (data not shown). We therefore normalized cytokine responses to WBC. This had little effect on the degree of inter-individual variation in cytokine concentrations. At the concentrations used, both bacterial ligands augmented median IL-1β, IL-6, TNF-α, G-CSF, IL-8, and IL-10 levels by two to four orders of magnitude above media values (Figure 1). However, relative augmentation of MCP-1 and IL-1ra was much less. 

**Figure 2:**
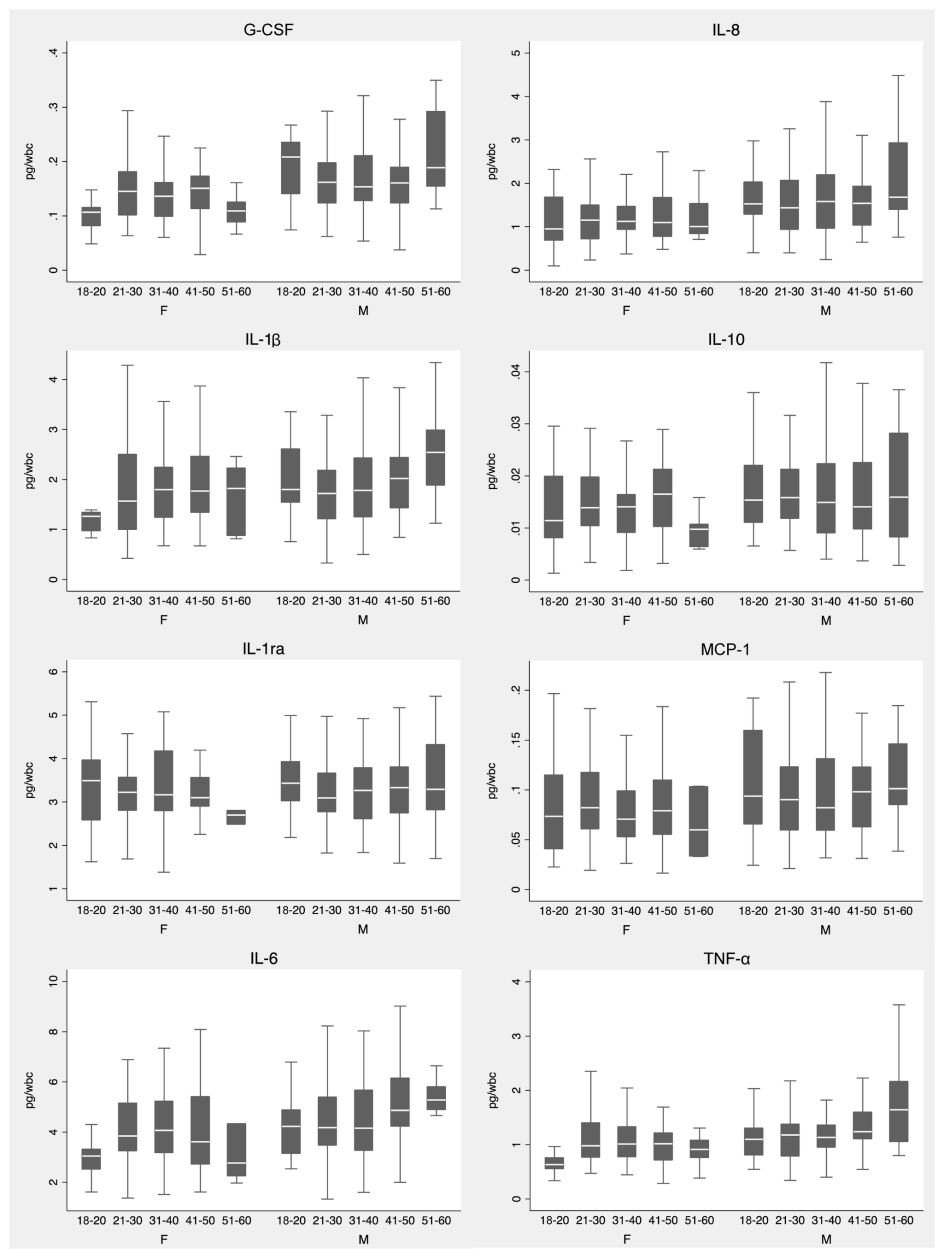
WBC-normalized plasma cytokine concentrations induced by stimulation of whole blood from 300 healthy subjects at 37°C for six hours with heat-killed *B. pseudomallei* 1026b 2.5 × 10^6^ CFU/ml, stratified by age and sex. Boxes show the median and interquartile range; whiskers show upper and lower adjacent values; outside values are not shown for clarity.

We next examined the effects of age and sex on the WBC-normalized cytokine response to *B. pseudomallei*. We observed that all concentrations of all cytokines except IL-1ra and IL-1β were significantly higher in males. We further observed that IL-6, TNF-α, G-CSF and IL-1β levels were positively associated with subjects’ age. We plotted cytokine responses to *B. pseudomallei* 1026b stratifying by age and sex (Figure 2), and noted that females tended to have lower cytokine responses at the extremes of age compared to the middle age range. In contrast, the oldest – and to a lesser extent the youngest – males tended to have higher cytokine responses than the middle age range. These data indicated the importance of considering age and sex in analysing inter-individual innate immune responses to *B. pseudomallei*.

**Figure 3:**
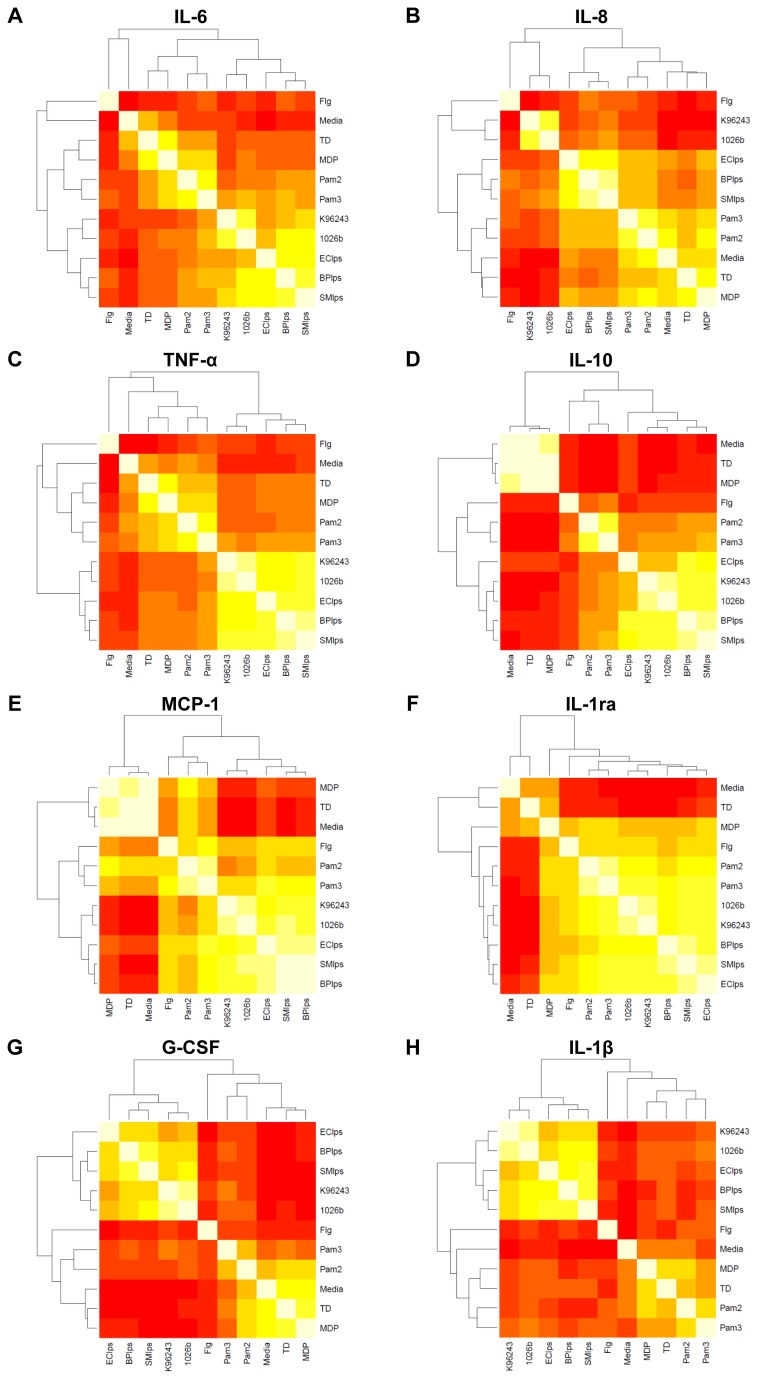
Heat map of correlation between cytokine concentration induced by stimulation of whole blood with a panel of purified innate immune ligands and heat-killed bacteria (as specified in the methods) with dendrogram of hierarchichal clustering results for the correlation matrix. Lowest correlation is red and highest correlation is white.

To further analyse the main drivers of the innate immune response to *B. pseudomallei* we examined the pairwise correlations between specific PAMPs and whole, heat-killed *B. pseudomallei*. For each of the eight cytokines assayed on the same platform, we created heat maps of the correlation between stimuli, including media, whole *B. pseudomallei* K96243 and 1026b, flagellin, Pam3CSK4, Pam2CSK4, MDP, TriDAP, *B. pseudomallei* K96243 LPS, *E. coli* O111:B4 LPS, and *S. minnesota* Re595 LPS (Figure 3). We found that the two *B. pseudomallei* isolates induced highly correlated levels of cytokines (all correlation coefficients > 0.8 and coefficients for TNF- α, MCP-1, IL-1ra, and IL-10 were ≥ 0.9). These observations demonstrated that the two *B. pseudomallei* isolates induced qualitatively similar innate immune responses.

We next assessed the degree of correlation between *B. pseudomallei* and the PAMPs *B. pseudomallei* LPS, *S. typhimurium* flagellin, Pam3CSK4, Pam2CSK4, MDP, or TriDAP. In comparison to all other stimuli, cytokine responses induced by *B. pseudomallei* were most highly correlated with responses induced by LPS. This was particularly marked for TNF-α, IL-10, MCP-1, G-CSF, and IL-1β (correlation coefficients for TNF-α shown in Table 1). Correlation between IL-8-induced by LPS and *B. pseudomallei* was lowest of any cytokine (r=0.49 and r=0.54 for K96243 and 1026b, respectively). Correlation between IL-1ra-induced by LPS and *B. pseudomallei* was highest of any cytokine (r=0.82 for both strains) but coefficients for IL-1ra responses induced by flagellin, Pam2CSK4, and Pam3CSK4 with *B. pseudomallei* were also greater than 0.70. Together, these observations suggested that the host inflammatory response to heat killed *B. pseudomallei* in this system is largely driven by LPS.

**Table 1:** Correlation (r) between human blood TNF-α response to whole heat-killed *B. pseudomallei* and to individual PAMPs.

	*B. pseudomallei* K96243	*B. pseudomallei* 1026b
*B. pseudomallei* LPS	0.78	0.78
*S. typhimurium* flagellin	0.30	0.28
Pam3CSK4	0.50	0.49
Pam2CSK4	0.40	0.41
MDP	0.38	0.40
TriDAP	0.36	0.37

**Figure 4:**
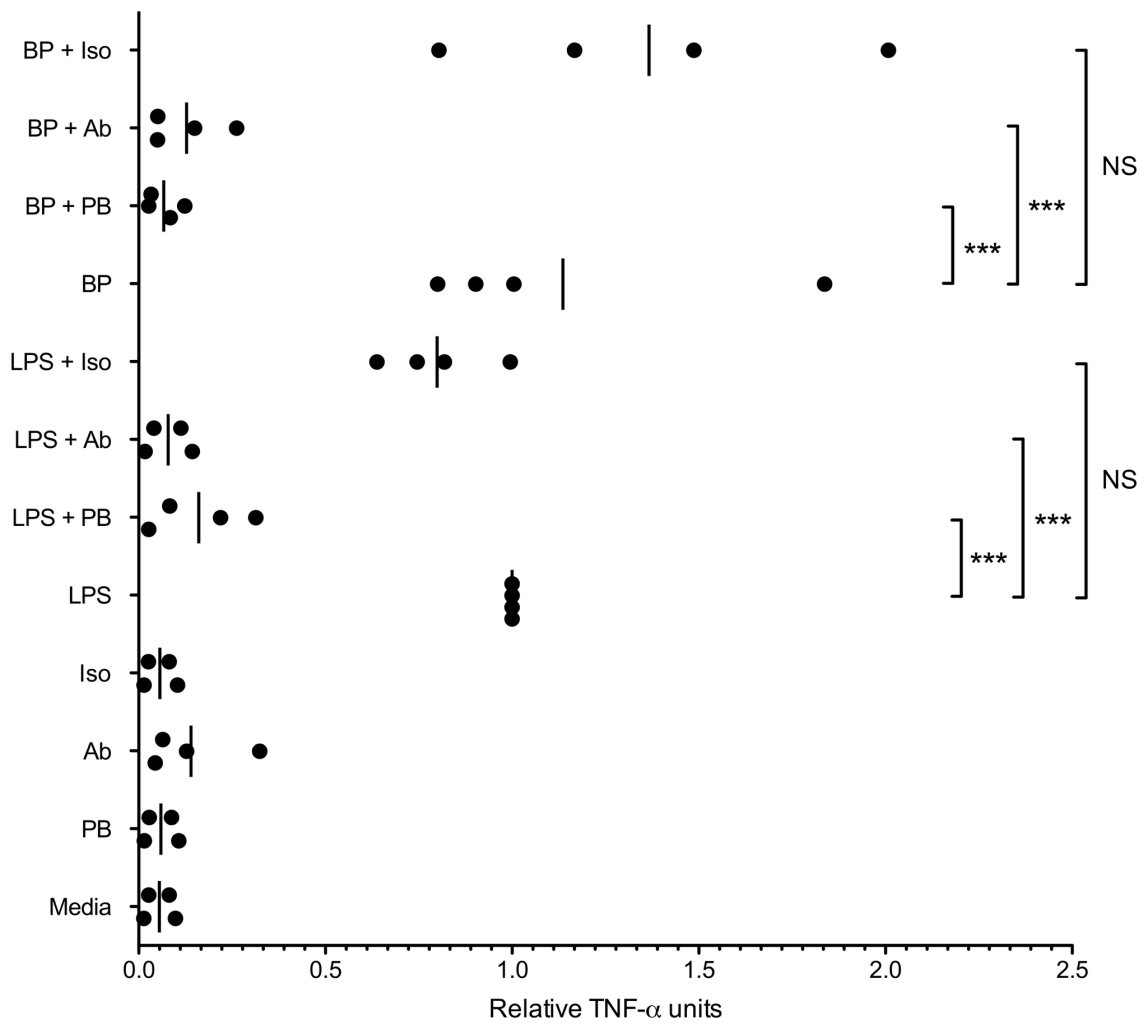
Blockade of the LPS-TLR4 axis markedly impairs TNF-α production induced by *B. pseudomallei* in human monocytes. Human monocytes (50,000/well) were stimulated with *B. pseudomallei* K96243 LPS 1 ng/ml (LPS) or heat-killed *B. pseudomallei* K96243 (bacteria:monocyte ratio of 1:1) (BP), with or without polymyxin B 100 μg/ml (PB) or a TLR4/MD2 neutralizing antibody 20 μg/ml (Ab) or isotype control 20 μg/ml (Iso). TNF-α was assayed in duplicate cell supernatants after 4 hours. Given substantial inter-individual variation in cytokine release, all TNF-α values for each individual were normalized to LPS-induced levels from the same subject. Relative TNF- α units for each individual (N=4) and mean values are displayed. Statistically significant differences were determined by ANOVA and a Bonferroni post-test. For clarity, only differences between each agonist alone (LPS or BP) and each agonist with polymyxin B or an antibody are shown. ***, p≤0.001. NS, p>0.05.

To more completely investigate this possibility, we designed an experiment in which human monocytes were stimulated with heat killed *B. pseudomallei* in the presence of inhibitors of the LPS-TLR4 axis. We treated monocytes with polymyxin B, an LPS antagonist [24] or with a TLR4/MD2 neutralizing antibody before stimulation with LPS or bacteria, and measured TNF-α release (Figure 4). As before, we found considerable inter-individual variation in cytokine response to or LPS or *B. pseudomallei*. We therefore normalized all TNF-α values for each individual to LPS-induced levels from the same subject. As expected, polymyxin B and the TLR4/MD2 neutralizing antibody inhibited LPS-induced TNF-α induction by an average of 84% and 92%, respectively. Polymyxin B and the TLR4/MD2 neutralizing antibody also markedly inhibited *B. pseudomallei*-induced TNF-α induction: by 93% and 86%, respectively. Thus, blockade of the PAMP LPS and of the PRR TLR4 had a profound effect on cytokine production induced by *B. pseudomallei* in human monocytes, further supporting a central role for LPS in the innate immune response.

*B. pseudomallei* LPS has been described as poorly stimulatory in comparison to other LPS isolates [11,12,14]. However, these experiments were performed in experimental animal models or in cell lines, rather than in human blood. We have shown that most *B. pseudomallei* LPS isolates are smooth; i.e. they have full-length O chains [25]. To compare the responses of human blood to various LPS isolates, we correlated cytokine responses induced by *B. pseudomallei* K96243 LPS (a smooth isolate) to responses induced by LPS from *E. coli* O111:B4 (smooth) and from *S. minnesota* Re595 (rough) in our study of 300 healthy subjects. Each LPS concentration was 10 ng/ml. *B. pseudomallei* LPS-induced cytokines were best correlated with *S. minnesota* LPS-induced cytokines (correlation coefficients ranged from 0.78 for G-CSF to 0.93 for MCP-1) but *B. pseudomallei* LPS-induced cytokines were also highly correlated with *E. coli* LPS-induced responses (for each cytokine correlation coefficients were at least 0.73 except for G-CSF which was 0.60). These data showed that there was substantial correlation in the blood cytokine response induced by LPS from *B. pseudomallei* compared to well-studied rough and smooth LPS isolates.

**Figure 5:**
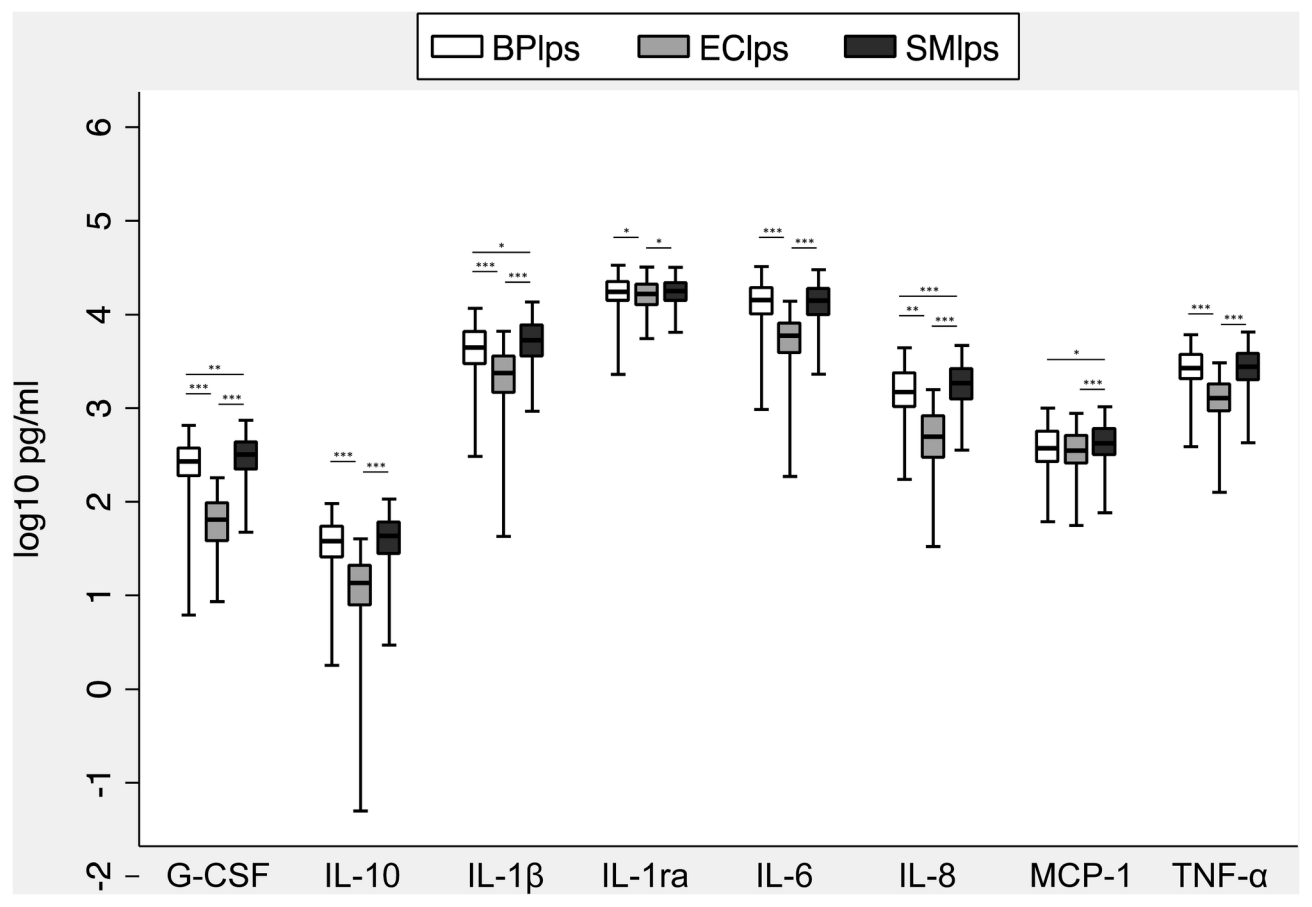
Plasma cytokine concentrations induced by stimulation of whole blood with *B. pseudomallei* K96243 LPS 10 ng/ml (BPlps), *E. coli* O111:B4 LPS 10 ng/ml (EClps), or *S. minnesota* Re595 LPS 10 ng/ml (SMlps). Boxes show the median and interquartile range; whiskers show upper and lower adjacent values; outside values are not shown for clarity. Statistical comparisons are made by ANOVA with a Bonferroni post test on log_10_ transformed data. *, p≤0.05; **, p≤0.01; ***, p≤0.001.

We next directly compared the magnitude of the whole blood cytokine response induced by 10 ng/ml LPS from *B. pseudomallei*, *E. coli*, and *S. minnesota* (Figure 5). At this concentration, we observed that *B. pseudomallei* LPS induced higher levels of all cytokines except MCP-1 than *E. coli* LPS and similar levels of IL-10, IL-1ra, IL-6, and TNF-α to *S. minnesota* LPS. A limitation to this comparison is that the endotoxic activity of LPS is attributable to the lipid A moiety [13] and given the variation in lipid A content between LPS isolates, comparing cytokine responses induced by comparable dry weights of different LPS isolates may not accurately reflect the potency of the lipid A of each LPS. We therefore used gas chromatography to determine the total nanomoles of lipid A fatty acids for each of the three LPS isolates. The amount of *E. coli*, *S.* minnesota, and *B. pseudomallei* lipid A fatty acids in each LPS preparation was 38.8, 363.4, and 31.7 nmol, respectively. Thus the amount of *B. pseudomallei* lipid A inducing the measured cytokine responses was similar to the amount of *E. coli* lipid A but was in fact ten fold less than the amount of *S. minnesota* lipid A. Together, these results indicated that, in comparison to two other LPS isolates, *B. pseudomallei* LPS induced at least similar – if not greater –levels of pro- and anti-inflammatory cytokines in human blood. 

## Discussion

*B. pseudomallei* is one of the most common bloodstream isolates identified in northeast Thailand [1,3]. Melioidosis – infection with *B. pseudomallei* – frequently manifests as sepsis, namely a marked host inflammatory response to infection [1,26]. Despite appropriate antibiotic use, the mortality rates in this setting are poor, averaging 40% over recent years [27]. Understanding the innate immune response in this disease may reveal new therapeutic strategies beyond traditional antimicrobials. Our large study of cytokine responses in human blood therefore provides important information derived from an at-risk population.

The most intriguing finding in this study is the significant role of *B. pseudomallei* LPS in driving the host response to *B. pseudomallei*, a result that contradicts existing beliefs. LPS has long been viewed as a key element in Gram-negative sepsis [10], although higher circulating levels of LPS have not been associated with greater mortality in melioidosis [28] and *B. pseudomallei* LPS is considered weakly inflammatory *in vitro* or in rabbits and mice [11,12]. In murine macrophages in vitro, *B. pseudomallei* LPS induces lower levels of pro-inflammatory cytokines and nitric oxide than *Salmonella abortus equi*, *E. coli*, or *Salmonella typhi* LPS isolates [11,12,14]. Reduced pro-inflammatory cytokine release induced by *B. pseudomallei* LPS compared to *E. coli* LPS in human THP-1 cells is also described [14]. Furthermore, the kinetics of TNF-α and NO release by *B. pseudomallei* LPS in murine macrophages are delayed [12]. 

There are several explanations for differences between our findings and previous reports. First, our studies focus exclusively on human whole blood responses rather than animal models, murine macrophages, or human macrophage-like cells. There may be important differences in the LPS-TLR4 axis between mice and humans in melioidosis: although we have found that human genetic variation in TLR4 is associated with susceptibility to melioidosis [16], in mice TLR2 seems to play a greater role than TLR4 in modulating the outcome of experimental *Burkholderia* infection [8,15,29]. In addition, the subjects recruited for our immuno-assay study were recruited from a melioidosis-endemic area, where the majority of the population is seropositive to *B. pseudomallei* by an early age [30]. The immune systems of these individuals may be primed to *B. pseudomallei* LPS and generate relatively higher inflammatory responses than to LPS from other pathogens. 

A second explanation for the discordance of our findings with prior studies is that each group used different LPS preparations and there is substantial variability in pathogenicity of different *B. pseudomallei* isolates [31] that may conceivably be attributable to LPS. We used K96243 because this is a fully sequenced reference strain originally obtained from a Thai patient [32]. It is also possible that different LPS isolation techniques altered the pro-inflammatory effects of the LPS. 

Our observation that TLR4/MD-2 blockade in human monocytes nearly completely abrogates *B. pseudomallei* LPS-induced pro-inflammatory cytokine production also provides additional confirmation that *B. pseudomallei* LPS triggers TLR4-dependent immune activation. We have previously shown that *B. pseudomallei* LPS is a human and murine TLR4 agonist [13]. This has been confirmed by others [14], although it has also been proposed that TLR2 is the *B. pseudomallei* LPS receptor [8]. Collectively, our findings challenge the prevailing belief that *B. pseudomallei* LPS is of lower pathophysiologic importance in human melioidosis and indicate that the LPS-TLR4 axis deserves further careful evaluation in this disease.

Our results also provide a novel overview of the cytokine response to *B. pseudomallei* in healthy Thai subjects. A surprising observation was the high baseline level of IL-1ra. However, this has also been demonstrated in healthy Caucasians [33], suggesting that this inhibitory molecule is constitutively expressed in a number of populations. We confirmed the presence in our population of substantial inter-individual variation, which has been reported by others and attributed to genetic variation [23,34]. Our findings of age- and sex-dependent variation in cytokine response to *B. pseudomallei* are of particular interest. We found that the inflammatory response to *B. pseudomallei* is higher in men than in women. This is concordant with previously published observations that LPS induces lower blood inflammatory responses in women than in men [35,36]. Male sex is a risk factor for melioidosis in northeast Thailand [3], although conceivably this is attributable to differential environmental exposure. However, the annual incidence of all-cause sepsis in the United States is also higher in men than in women [37]. Among culture-proven cases with melioidosis, sex is not associated with mortality [5]. Although innate immune responses are blunted in the very young and old [38], we further identified a novel pattern of responses that differed between males and females across a more modest age range. Female responses tended to be lower in individuals aged 18-20 and 51-60, whereas male responses tended to increase in these same ranges. Future clinical studies will be required to determine how these *ex vivo* differences translate into outcomes. 

Our study has several strengths: rigorous selection of healthy subjects from a single location, a large sample size, and standardized, batched preparation of immuno-assay plates using two well-described strains of *B. pseudomallei* and a panel of pathogen-associated molecular patterns, and immediate stimulation of blood after collection. These elements reduced experimental variability and enhanced our ability to interpret the data. Several studies to date have yielded valuable information about the human innate immune response in clinical melioidosis [8,39–44]. While reflecting the realities faced by a clinician, a limitation of such studies is the often unknown variation in route of infection, in timing of infection and presentation for clinical care, in treatments administered, and in co-morbid conditions. Animal models of infection are widely used and have numerous benefits; however, they may not optimally reflect human sepsis [45–47]. Faced with these challenges, our study sought to better characterize the human innate immune response to *B. pseudomallei* by stimulating whole blood from carefully selected subjects under highly controlled conditions. 

A potential limitation to our study is that we used heat-killed, rather than live, bacteria. As our study focused on PAMPs present in live or dead bacteria, this is unlikely to significantly alter our results. Moreover, given the biosafety and biosecurity restrictions on use of live *B. pseudomallei* in the US and in Thailand this would have been prohibitively complex. Although lipid A is the pro-inflammatory component of LPS, it is possible that the use of basic 0.2% triethylamine in our LPS extraction procedure may have altered the O-antigen structure in the LPS preparations studied. While our data is suggestive of the role of LPS in human melioidosis, we cannot directly extrapolate from our experimental *ex vivo* system to *in vivo* infection. Diabetes alters the immune response to infection and diabetes is the major risk factor for melioidosis; future studies should therefore test whether the present observations are also noted in diabetic subjects.

In conclusion, we report the largest assessment of the human whole blood innate immune response to *B. pseudomallei*, identify age- and sex-dependent variation, and demonstrate a previously unsuspected role for *B. pseudomallei* LPS as a driver of the response.
